# Predictive analysis of metabolic syndrome based on 5-years continuous physical examination data

**DOI:** 10.1038/s41598-023-35604-8

**Published:** 2023-06-05

**Authors:** Guohan Zou, Qinghua Zhong, Ping OUYang, Xiaoxi Li, Xiaoying Lai, Han Zhang

**Affiliations:** 1 School of Physics and Telecommunication Engineering, South China Normal University (SCNU), Guangzhou, 510000 China; 2 School of Electronics and Information Engineering, SCNU, Foshan, 528225 China; 3 Guangdong Provincial Engineering Technology Research Center of Cardiovascular Individual Medicine & Big Data, SCNU, Guangzhou, 510006 China; 4 Department of Health Management, Nanfang Hospital, Southern Medical University, Guangzhou, 510515 China

**Keywords:** Diseases, Endocrinology, Health care, Medical research, Risk factors

## Abstract

Metabolic syndrome (MetS) represents a complex group of metabolic disorders. As MetS poses a significant challenge to global public health, predicting the occurrence of MetS and the development of related risk factors is important. In this study, we conducted a predictive analysis of MetS based on machine learning algorithms using datasets of 15,661 individuals. Five consecutive years of medical examination records were provided by Nanfang Hospital, Southern Medical University, China. The specific risk factors used included WC, WHR, TG, HDL-C, BMI, FGLU, etc. We proposed a feature construction method using the examination records over the past four consecutive years, combining the differences between the annual value and the normal limits of each risk factor and the year-to-year variation. The results showed that the feature set, which contained the original features of the inspection record and new features proposed in this study yielded the highest AUC of 0.944, implying that the new features could help identify risk factors for MetS and provide more targeted diagnostic advice for physicians.

## Introduction

Metabolic syndrome (MetS) is a pathological state in which the patient suffers from metabolic disorders of proteins, fats, and carbohydrates^1^. It is mainly characterized by hypertension, hyperglycemia, obesity, and dyslipidemia^2–4^. Over the past three decades, the number of MetS patients has increased^5–7^, which has resulted in the rise of related diseases such as diabetes, cardiovascular diseases, and cancer^8–12^. The prevalence of MetS among Chinese adults is about 11.0%, making it a significant public health problem in China^13^. Therefore, researchers need to focus on disease prevention, diagnosis, and intervention to reduce the risk factors that increase with metabolic disorders and prevent them from further developing into other more serious diseases, which might impose a greater physical burden on patients.

Many studies have investigated ways to identify MetS at an initial stage with high accuracy. Risk factors associated with the morbidity of MetS, such as triglycerides (TG), high-density lipoprotein cholesterol (HDL-C), white blood cells (WBCs) and related subtypes, and alanine aminotransferase (ALT), have been recognized^14–16^. Some studies have investigated changes in anthropometric features such as the body mass index (BMI), waist circumference (WC), and waist-to-hip ratio (WHR)^17,18^. However, these approaches did not incorporate the latest machine learning algorithms into their models, which limited the accuracy and interpretation of their predicted outcomes.

Machine learning techniques are frequently used in healthcare. They can be used to efficiently extract information from big data to aid decision-making and help in understanding the nonlinear and complex relationships among various factors, thus significantly improving diagnostic accuracy^19,20^. Many researchers have used machine learning algorithms to develop models for predicting MetS. Edelenyi et al. used random forests to predict the status of MetS^21^, and their data were obtained from a large case-control study that contained reliable records of genetically relevant parameters and dietary intake. They found that machine learning techniques can be used to effectively predict the emergence of MetS several years in advance. For example, a specific imbalance in the composition of plasma fatty acid revealed the risk of developing MetS. Worachartcheewan et al. used random forests to identify important risk factors and to predict the risk of MetS in a Thai population^22^. In their study, the model with 40 trees had higher confidence than the model with 20 trees. Their results indicated that the TG level was the most critical health parameter associated with MetS. Farzaneh Karimi-Alavijeh et al. used decision trees and the support vector machine method (SVM) to predict the 7-year incidence of MetS^23^, where the sensitivity, specificity, and accuracy of SVM, were found to be 0.774 (0.758), 0.74 (0.72) and 0.757 (0.739), respectively. The results showed that the sensitivity, specificity, and accuracy of SVM approach were more effective than that of decision trees, and TG, blood pressure (BP), and BMI were found to be the most important features for predicting MetS. Kyung Choe et al. used machine learning models of genetic and clinical information from a non-obese healthy population for predicting MetS^24^, and used five machine learning approaches simultaneously. They found that naive Bayesian classification the best performance (AUC = 0.65).

The results of these experiments suggested that machine learning techniques, especially nonlinear classifiers such as gradient-enhanced trees and random forests, generally predict clinical outcomes more accurately than traditional statistical methods. However, these studies mostly used data on basic anthropometric features that were obtained directly from data sources, without combining information on the difference between the value and the normal limits and time-series changes over the years. A set of relevant features should be selected that can comprehensively describe all concepts in a given dataset. Yang et al. extracted differential numerical features and differential state features (DNFs and DSFs) in consecutive multi-year datasets to investigate the effect of temporal changes in the value and state of indicators on the risk of prevalence of MetS^25^. However, information on differential features only reflects the year-to-year trend, more information is not combined to know the difference in the risk of disease when risk factors change in different specific values every year. Therefore, this method does not fully elucidate the development of risk factors.

To address the above limitations, we used individual continuous physical examination datasets of 15,661 subjects recorded over five consecutive years to construct a new feature, known as the differential fluctuant feature (DFF), for predicting the presence of MetS and investigating the effect of the difference between the value and the normal limits of each risk factor, as well as the temporal variation in the risk of MetS. Our findings indicated that crucial risk factors such as TG, WC, BMI, and HDL-C, which include the original values derived from the most recent 1-year examination records or the corresponding values of each DFF, were significant predictors for the risk of MetS.

## Results

### Performance of different feature sets

To visualize whether the DFFs proposed in this study had performance advantages, we added DNFs and DSFs proposed by^25^ and DFFs to the feature set containing the original features of the past 5 years to compare the performance of different classifiers and feature set combinations. We performed 10-fold cross-validation experiments, and expressed the AUC as the mean ± standard deviation. The results for the XGBoost classifier are presented in Table 1, and the results with the best average validation metric are highlighted in bold. The machine learning process was performed in Python using scikit-learn, and the plots were generated using matplotlib^26,27^.

**Table 1:** Results based on the XGBoost model with different feature sets. The results with the best performance in each metric are marked in bold characters.

Feature Set	AUC	Accuracy	Sensitivity	Specificity	Precision	F1-score
Original features	0.904(±0.008)	0.873(±0.012)	0.738(±0.059)	0.884(±0.007)	0.378(±0.054)	0.500(±0.034)
With DNFs & DSFs	0.924(±0.010)	0.883(±0.012)	0.768(±0.043)	0.892(±0.007)	0.402(±0.047)	0.524(±0.039)
With DFFs	**0.944(± 0.008)**	**0.906(± 0.008)**	**0.786(± 0.036)**	**0.918(± 0.008)**	**0.498(± 0.029)**	**0.592(± 0.032)**

The AUC results obtained from the machine learning prediction model improved to varying degrees upon integrating DSFs and DNFs, and incorporating DFFs, which suggested that incorporating additional features can improve the performance considerably. The feature set with DFFs performed significantly better in AUC than DSFs and DNFs, which supported the effectiveness of DFFs for predicting MetS in the following year.

The cross-sectional comparison of various classifiers showed that all three machine learning classifiers used in this study, included XGBoost, Random Forest, and Stacking, significantly outperformed the conventional LR classifier. Additionally, XGBoost emerged as the optimal choice with the best results. Hence, unless stated otherwise, we used XGBoost as the classifier for subsequent experiments.

**Figure 1:**
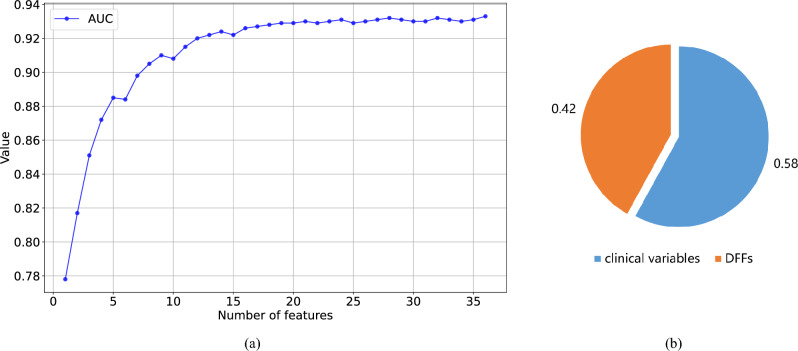
(**a**) The AUC variation of the increase in the numbers of features based on the XGBoost model. (**b**) The proportion of the importance of clinical variables and DFFs based on the XGBoost model.

**Figure 2:**
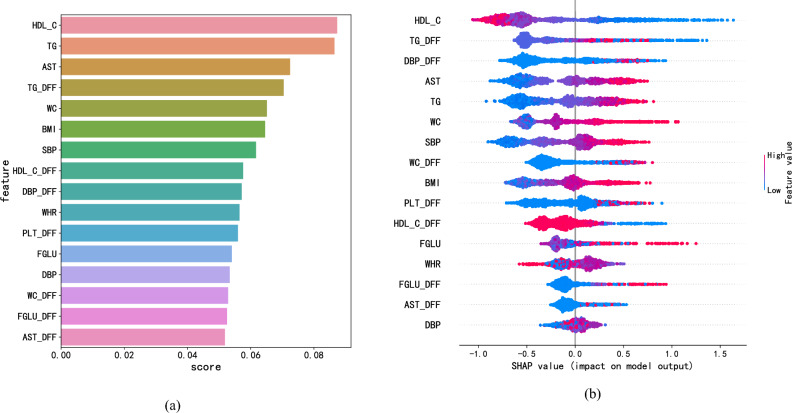
(**a**) The feature importance based on the XGBoost model. AST, aspartate aminotransferase; SBP, systolic blood pressure; DBP, diastolic blood pressure; PLT, platelets; FGLU, fasting plasma glucose. (**b**) The feature importance based on the SHAP model.

### Risk factors associated with MetS

The feature importance ranking graph obtained by XGBoost allowed us to select the MetS risk factors with the top scores higher rank in the graph indicated that the feature affected the MetS risk prediction more significantly. Since DFF already contained information on the changes in the corresponding original values over the past years, we only screened the original features in the most recent year and DFFs for the analysis. We also increased the number of features one by one based on the importance scores of the above features, starting with the features with the highest scores. The number of features to be analyzed was determined according to their AUC convergence changes. As shown in the AUC change curve in Fig. 1a, after the number of features increased to 16, the growth rate of AUC decreased and gradually converged to 0.930. Hence, we selected the top 16 features as significant risk factors, and the final plotted feature importance ranking is shown in Fig. 2a.

These 16 features were divided into the clinical variables of the most recent year and the DFFs associated with them. The clinical variables included HDL-C, TG, AST, BMI, WC, SBP, DBP, WHR and FGLU, and DFFs included TG_DFF, DBP_DFF, WC_DFF, PLT_DFF, HDL-C_DFF, AST_DFF and FGLU_DFF. The DFFs comprised nearly half of the top 16 critical features, and the importance of these seven new features accounted for 42% of the overall features, as shown in Fig. 1b, which indicated that the method was quite robust.

To further analyze the contribution of the top 16 features to the prediction of MetS, we performed an interpretive analysis using the SHAP tool^28^. As shown in Fig. 2b, the new features, including TG_DFF, DBP_DFF and WC_DFF, contributed the most to the prediction of MetS, and HDL-C, AST and TG were the top three most critical features among the original features. For TG, the original and new features significantly increased the risk of MetS. The original and new features of AST were found to be essential for increasing the risk. As only a few studies have investigated whether AST is a significant risk factor for MetS, this factor needs to be further investigated.

Next, we analyzed the effect of abnormalities in important original features, especially DFFs on patients of different genders and age groups (classified by the World Health Organization) to further compare the original features and DFFs.

### Impact of original features on the risk of MetS

When the value of each original feature exceeds the normal limit, the feature is considered to be in an abnormal state. We first calculated the odds ratio (OR) of the abnormality of each original feature related to the risk of MetS in the following year and analyzed the performance of the above features in both sexes and different age groups. The OR performance in males is presented in Table 2. The abnormal performance of TG and HDL-C was recorded in males of all age groups. The abnormal performance of the BMI was found to increase the risk of disease in males who were below 60 years, and the risk of disease in older males was more, as determined by the abnormal performance of AST. The OR performance of the abnormality of each original feature in females is presented in Table 3. The abnormalities in TG, WC, and FGLU were considerable among young women. The most important risk factors for females aged 45–59 years were TG,TG, HDL-C and BMI, while the most important risk factors for older females were AST, BMI and FGLU.

**Table 2:** The OR performance of each original feature’s abnormality to MetS by age groups in males. The result with the best performance in each metric are marked in bold characters.

Feature	Age 18–44 (95%CI)	Age 45–59 (95%CI)	Age >=60 (95%CI)
HDL-C	**3.710 (3.072–4.480)**	**3.585 (2.662–4.829)**	**2.662 (1.515–4.679)**
TG	**5.216 (4.411–6.168)**	**4.194 (3.311–5.313)**	**2.571 (1.576–4.195)**
AST	2.938 (1.996–4.323)	1.837 (0.982-3.437)	**2.828 (1.092–7.326)**
BMI	**3.626 (3.050–4.311)**	**2.486 (1.961–3.151)**	2.430 (1.659–3.559)
WC	2.295(1.878–2.805)	1.758 (1.337–2.312)	2.274 (1.437–3.600)
SBP	1.843 (1.351–2.514)	1.989 (1.483–2.668)	1.722 (1.180–2.515)
DBP	2.231 (1.644–3.028)	1.605 (1.155–2.230)	1.256 (0.720–2.192)
WHR	2.126 (1.810–2.498)	1.704 (1.355–2.142)	2.306 (1.573–3.382)
FGLU	3.298 (1.792–6.072)	1.589 (0.950–2.659)	1.112 (0.575–2.149)

**Table 3:** The OR of original feature’s abnormality to MetS by age groups in females. The results with the best performance in each metric are marked in bold characters.

Feature	Age 18–44 (95%CI)	Age 45–59 (95%CI)	Age >=60 (95%CI)
HDL-C	7.851 (3.990 15.446)	**7.073 (3.103 16.121)**	3.222 (2.830–3.903)
TG	**10.207 (6.056–17.201)**	**6.397 (3.937–10.394)**	3.414 (1.653–7.051)
AST	2.831(1.983–3.990)	**7.951 (2.808–22.513)**	**9.366 (2.023–43.352)**
BMI	9.947 (5.875–16.841)	4.052 (2.504–6.556)	**4.659 (2.416–8.988)**
WC	**10.042 (5.739–17.571)**	5.178 (3.121–8.592)	2.794 (1.247–6.259)
SBP	3.275 (1.164–9.214)	1.594 (0.823–3.087)	1.334 (0.695–2.560)
DBP	5.961 (2.312–15.365)	2.694 (1.187–6.114)	2.209 (0.432–11.300)
WHR	4.563 (2.650–7.859)	4.049 (2.357–6.957)	3.141 (1.460–6.759)
FGLU	**13.737 (5.121–36.852)**	5.322 (2.383–11.886)	**3.978 (1.579–10.024)**

Combining the results presented in Tables 2 and 3, we found that most of the risk factor abnormalities had a significantly higher risk of developing the disease in younger people than in middle-aged and older people. The risk was higher in females than in males of the same age group. These results were similar to those of other studies^25,29,30^.

### Impact of DFFs on the risk of MetS

Initially, we defined the normal and abnormal states of DFFs by calculating discrete values. The state was considered normal when the absolute value was lower than the mean value and abnormal when it was higher than the mean value.

**Figure 3:**
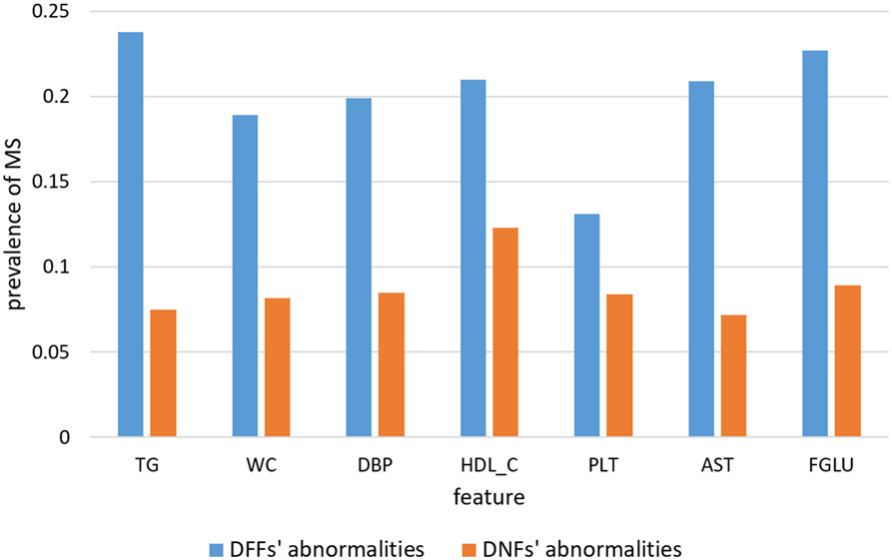
The prevalence of MetS with the value of DFFs and DNFs in the abnormal state.

Figure 3 depicted the prevalence of MetS with each DFF in the abnormal state under the above differentiation and compared the prevalence of MetS with each DNF in the abnormal state. DNF was considered to be abnormal when its value was higher than 0. The prevalence of MetS with DFFs was significantly higher than that with DNFs, which indicated that DFFs could more effectively distinguish between people who have greater chances of developing MetS in the coming year. The highest prevalence (0.24) was observed when TG_DFF exceeded the mean value, followed by the prevalence of FGLU_DFF (0.23). The value of DFF reflected the magnitude of the change over the past 4 years. The larger the value of DFF, the greater the magnitude of the change and the higher the prevalence of MetS.

We also evaluated the effect of abnormalities in DFF on the occurrence of the disease in the coming year, as shown in Tables 4 and 5. We found that TG_DFF abnormalities increased the risk of disease in all males. Additionally, DBP_DFF abnormalities increased the risk of disease in middle-aged males, and WC_DFF abnormalities increased the risk of disease in young and older males. In females, WC_DFF abnormalities increased the risk of disease under the age of 60 years, and FGLU_DFF and TG_DFF abnormalities significantly increased the risk in young and middle-aged females. Older females had a higher risk of AST_DFF and FGLU_DFF abnormalities, which significantly increased the risk of disease.

**Table 4:** The OR of DFFs’ abnormality to MetS by age groups in males. The results with the best performance in each metric are marked in bold characters.

Feature	Age 18–44 (95%CI)	Age 45–59 (95%CI)	Age >=60 (95%CI)
TG_DFF	**5.220 (4.420–6.165)**	**3.843 (3.043–4.854)**	**2.960 (1.956–4.479)**
DBP_DFF	2.527 (1.994–3.202)	**2.293 (1.765–2.979)**	1.612 (1.064–2.441)
WC_DFF	**2.733 (2.263–3.300)**	2.039 (1.584–2.625)	**2.584 (1.675–3.988)**
PLT_DFF	2.591 (2.073–3.238)	2.166 (1.566–2.994)	2.366 (1.325–4.227)
HDL-C_DFF	2.071 (1.749–2.453)	1.541 (1.191–1.994)	2.389 (1.498–3.811)
AST_DFF	2.534 (2.035–3.157)	2.129 (1.502–3.018)	0.931 (0.459–1.887)
FGLU_DFF	2.143 (1.469–3.127)	1.956 (1.392–2.748)	2.398 (1.553–3.703)

**Table 5:** The OR of DFFs’ abnormality to MetS by age groups in females. The results with the best performance in each metric are marked in bold characters.

Feature	Age 18–44 (95%CI)	Age 45–59 (95%CI)	Age >=60 (95%CI)
TG_DFF	5.488 (3.229– 9.326)	**10.077 (6.126–16.575)**	2.514 (1.299–4.863)
DBP_DFF	6.290 (3.031–13.057)	2.491 (1.332–4.656)	4.035 (1.994–8.169)
WC_DFF	**11.204 (6.640–18.905)**	**4.123 (2.508–6.779)**	1.854 (0.926–3.712)
PLT_DFF	3.378 (2.037–5.601)	3.164 (1.939–5.163)	2.024 (0.923–4.435)
HDL-C_DFF	2.840 (1.527–5.283)	3.229 (1.681–6.204)	0.639 (0.080–5.112)
AST_DFF	1.953 (0.603–6.323)	2.149 (.954–4.840)	**4.090 (1.761–9.500)**
FGLU_DFF	**6.807 (3.151–14.703)**	2.912 (1.484–5.715)	**4.696 (2.259–9.766)**

## Discussion

In this study, we used longitudinal data recorded over five consecutive years to construct a new feature set for examining the risk prediction of MetS. By integrating the difference between the annual value and the normal limits of each risk factor with the year-to-year variation, we achieved a more comprehensive understanding of the development of risk factors. We evaluated various classifiers on different feature sets using the ten-fold cross-validation method to assess their discriminative performance. We found that XGBoost outperformed other classifiers, and we recorded a maximum AUC of 0.944 by incorporating the original features and DFFs. We compared our approach to that of other studies in this area and found that our results were better. We also found that DFFs can perform better by capturing the nonlinear interactions between the features and the target variable.

We statistically analyzed the abnormal performance of 16 critical indicators for different sex and age groups of MetS based on the feature importance ranking results of the best-performing XGBoost classifier. The results showed that the diseased and average populations differed in the abnormal performance of single feature values.

Among the original features, the impact of the abnormalities in TG, HDL-C, AST, and BMI was prominent in males and females, and many other studies have shown that these are important risk factors. After analyzing the original features, we constructed DFFs, assigned abnormal and normal states based on their numerical results, and then, statistically analyzed the numerical results of DFFs from several perspectives. Our results showed that when the value of a single DFF exceeded the mean value, the prevalence of risk factors in the corresponding population increased. The highest prevalence was observed for TG_DFF, followed by FGLU_DFF, which implied that high values of TG_DFF and FGLU_DFF were the most important characteristics for prevalence. The prevalence of MetS with abnormal DFFs was significantly higher than that of abnormal DNFs, which indicated that DFFs can more effectively highlight the affected population. We also calculated the OR values of DFFs. Specifically, abnormal TG_DFF increased the risk of disease in all males, which matched the results for the original TG described above, indicating that abnormal TG increased the risk most significantly in males. Abnormalities in WC_DFF increased the risk among women under 60 years, and AST_DFF and FGLU_DFF were more significant risk factors in older females. AST as a risk factor was also present in the original index, and the effect of AST on the disease in the elderly group was prominent. This factor should be further studied as reports on it are rare.

To summarize, in this study, we evaluated new features using medical data collected over five consecutive years. We found an association between the onset of MetS and specific clinical variables, including the prevalence of the disease and OR performance in different age and sex groups. The results showed that the performance of new features can effectively distinguish between the diseased and healthy populations, and it can help understand the relationship between lifestyle and the pathogenesis of MetS.

This study had some limitations. First, all samples in the dataset of this study were from Guangdong Province, China, and thus, the experimental results might have regional characteristics. Second, there might be better formulae for replacing DFFs proposed in this study, and we aim to continue searching for more reasonable and effective new feature construction methods in subsequent studies to more comprehensively describe the development of risk factors.

## Methods

### Diagnostic criteria for MetS

Many researchers around the world have developed clinical criteria for MetS^31–34^. To avoid inconsistencies caused by various criteria and best fit the local context of the experimental data, we used the criteria proposed by the Chinese Guidelines for the Prevention and Treatment of Type 2 Diabetes (2017 edition) in this study to identify patients with MetS.

According to the guidelines, patients with MetS can be diagnosed by meeting three or more of the following five conditions.

1. Abdominal obesity: WC>= 90/85 cm (male/female).
2. Hyperglycemia: fasting glucose (FGLU)>= 6.1 mmol/L or 2-h postprandial glucose (PG)>= 7.8 mmol/L and/or previously diagnosed and treated diabetes mellitus.
3. Hypertension: BP>= 130/85 and previously diagnosed and treated hypertension.
4. Fasting TG>= 1.70 mmol/L.
5. Fasting HDL-C<= 1.04 mmol/L.

### Datasets

We used medical checkup data provided by the Health Management Department of Southern Hospital of Southern Medical University. The dataset contained 1,039,564 medical checkup records for 546,918 participants across 21 prefecture-level cities and subordinate districts and counties in South China, including Guangzhou, Foshan, etc. The inclusion criteria targeted individuals who were 18–80 years old, based on continuous physical examinations taken from 2009 to 2019.

The hospital staff collected numerous raw indicators, including anthropometric data, blood parameters, other biochemical indicators, medical history, gender and age, by extracting values recorded in the physical examination report. Based on the data provided by the hospital, we first derived two additional characteristics from the available anthropometric variables, including the waist-to-hip ratio (WHR) and the body mass index (BMI). Since we aimed to extract new features from specific indicator values that can reflect temporal changes, we extracted 18-dimensional continuous-type numerical features from the data.

After determining the features to be extracted, we cleaned the dataset. We first excluded individuals who underwent too many physical examinations (greater than 20). We then removed outliers for each indicator, including abnormal records for age (greater than 80 years), based on the upper and lower limits of the indicators, as determined by the physicians. After removing the outliers, the treatment of missing values was equally important, as too many missing values can make the model complex. We used different padding strategies for the missing rate, data type and value distribution of each indicator. If the number of missing values was large (more than 70% of data was missing), the feature was deleted. For features with a small number of missing values, we used mean padding if the feature obeyed a normal distribution and median padding if it obeyed a skewed distribution.

After pre-processing the data, we obtained usable structured data containing 530,091 male patients and 398,793 female patients. A more detailed description of these extracted features is shown in Table 6.

This study was approved by the Academic Committee of South China Normal University (Approval No.: SCNU-PHY-2020-063). All methods we used in the study adhered to relevant ethical guidelines and regulations (the Declaration of Helsinki). All patients signed an informed consent form before their data were included in the study.

**Table 6:** Description of the raw data set. The characteristics are expressed as mean ± standard deviation. LDL-C, low-density lipoprotein cholesterol; TC, total cholesterol; UA, uric acid; HGB, hemoglobin; RBC, red blood cell; CR, creatinine.

Feature	Male(N=530091)	Female(N=398793)
Age (year)	39.65 ± 13.33	37.18 ± 13.08
WC (cm)	82.52 ± 8.12	75.35 ± 7.74
WHR	0.89 ± 0.08	0.82 ± 0.08
DBP (mmHg)	74.95 ± 10.11	70.19 ± 8.98
SBP (mmHg)	123.47 ± 14.23	115.87 ± 14.35
TG (mmol/L)	1.65 ± 1.43	1.14 ± 0.72
HDL-C (mmol/L)	1.29 ± 0.24	1.43 ± 0.28
LDL-C (mmol/L)	3.11 ± 0.60	2.95 ± 0.55
BMI (kg/m⌃2)	24.03 ± 3.05	22.31 ± 2.82
TC (mmol/L)	5.20 ± 0.91	5.04 ± 0.85
FGLU (mmol/L)	5.00 ± 1.10	4.86 ± 0.78
UA ($\mu$mol/L)	403.71 ± 81.79	314.65 ± 66.60
ALT (U/L)	25.99 ± 14.92	16.08 ± 8.80
AST (U/L)	24.02 ± 7.91	20.13 ± 6.06
HGB (g/L)	151.97 ± 10.64	133.97 ± 11.82
RBC count (10⌃12/L)	5.15 ± 0.47	4.65 ± 0.40
WBC count (10⌃9/L)	6.72 ± 1.54	6.28 ± 1.39
PLT (10⌃9/L)	239.78 ± 48.54	256.88 ± 53.47
CR ($\mu$mol/L)	77.63 ± 15.18	58.15 ± 13.67

### The MetS prediction model

This study was conducted to compare the differential performance of changes in indicators between those who developed a disease from a healthy state and those who remained healthy. Our findings might help in the effective prevention and intervention of the physically examined population for MetS-related risk factors. We considered the results of five consecutive years of physical examinations for each individual. The features extracted from the multi-year records were used as input to identify the features that could represent physical and physiological changes in the body over time.

**Figure 4:**
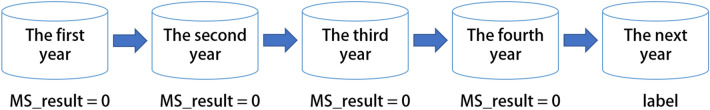
The schematic diagram of the MetS risk prediction model within the next 1 year.

The prediction can be considered to be supervised classification, and the first four records used as input in the constructed 5-year model were the features under the healthy state (MS_result = 0). The patients suffering from MetS in the following year were marked as 1 or 0. Therefore, one sample in the model had data on multiple consecutive physical examinations, representing the numerical situation of each index in each year, as shown in Figure 4. After constructing the model, we obtained 15,661 valid samples, of which 1338 and 14,323 samples suffered and did not suffer from MetS in the following year, respectively.

### Feature structure

Firstly, we described the year-to-year variation of the indicator values. In this case, the numerical difference feature (DNF) was represented as

1 $$\begin{aligned} {I\_DNF = I_2 - I_1} \end{aligned}$$

where $I_2$ and $I_1$ represent the specific values of the indicator in the current year and the past year, respectively. Thus, $I\_DNF$ could describe the absolute value change of the indicator with the year.

However, it was not enough to have numerical differential features to describe the variation of features in time series. When a patient and an average person changed the exact value of an indicator simultaneously, their importance differed. Therefore, we introduced a weighting function that reflected the different importance when different values were brought to change based on different values.

The weighting function has the following main requirements.

1. The indicator produces changes in different values, and the risk weight it imposes are different.
2. The higher the value of each indicator, the higher the risk when generating changes, i.e., the risk weight is an increasing function that grows with the value.
3. The fastest growth rate of risk is when qualitative changes occur around the upper and lower limits of the indicator.

We obtained by observing different function algorithms that the underlying formula of the Sigmoid function meets our requirements. The image of the Sigmoid function is continuous and smooth, strictly monotonic, and symmetric with a (0,0.5) center. Therefore, the risk growth curve could be described as

2 $$\begin{aligned} {S(x) = 1 / (1 + e^{-ax})} \end{aligned}$$

where *x* is the difference between the current indicator value and the upper limit of the normal range of that indicator, and *a* is a function parameter.

According to the above formulas, the new feature was defined as the product of the numerical change weight function and the numerical difference feature of the indicator, as shown in

3 $$\begin{aligned} {F(x) = I\_DNF * S(x)} \end{aligned}$$

where the function *S*(*x*) is a weight function modeled after the Sigmoid function, and $I\_DNF$ is the difference between the values for a certain 2-year period. The result is expressed as the effect of the disease brought about by a specific 2-year change at different values.

Since the study was conducted on a sample set of consecutive years, we obtained three new features for each indicator, i.e., new features generated by the above formula for years 1-2, 2-3, and 3-4. To effectively combine the three time periods, we gave different weights to the new features for each period, where the new features closer to the current time have higher weights, which were determined using the formula $b^3 +b^2 +b^1=1$. Finally, the formula of new features was shown as

4 $$\begin{aligned} {G(x_1,x_2,x_3)=b^3*I_1\_DNF*S(x_1)+b^2*I_2\_DNF*S(x_2)+b*I_3\_DNF*S(x_3)} \end{aligned}$$

where $x_1$, $x_2$ and $x_3$ are the differences between the values and the normal limit in the past every year. Moreover, $I_1\_DNF$, $I_2\_DNF$ and $I_3\_DNF$ are the differences between the values in years 1-2, 2-3, and 3-4.

We reconciled parameters a and b in subsequent work based on cross-validation of machine learning to achieve optimal performance. Since each indicator had taken different value ranges, parameter *a*’s significance was to scale the value ranges of different indicators uniformly. Therefore, we normalized the maximum value of *x* by (1/maximum value of different indicators) and then gave the parameter value $a=50$ which performed best in machine learning according to different scaling degrees. For parameter *b*, the optimal parameter $b=0.6$ was selected based on the exact weight formula as above with the performance of machine learning.

### Machine learning classifier

We used several algorithms to observe the performance of predictive models in machine learning for 5 years of data, including the more commonly used machine learning algorithms and the traditional logistic regression algorithm.

1. XGBoost: XGBoost consists of several decision trees, of which the decision tree is a CART regression tree model. The main idea of this classifier is to continuously learn new regression trees to fit the residuals of the last prediction, thus obtaining very high accuracy.
2. Random Forest (RF): RF is a common machine learning classifier based on the bagging algorithm^35^, which also consists of a combination of multiple decision trees. Compared with the traditional single-tree classifier, RF has a fairly high-performance optimization.
3. Stacking: Stacking is an integrated classifier based on adding another layer of classifiers on top of the original classifier, then selecting the target labels predicted by most of the classifiers by a voting method. In this paper, we let Stacking combine XGBoost and RF.
4. Logistic Regression (LR): LR is a traditional classical classifier, which works similar to linear regression, assuming that the data obeys a certain distribution and then using a great likelihood estimation algorithm to do parameter estimation.

### Evaluation

In our experiments, the primary metric used to determine the classifier’s performance was the Area Under The Curve (AUC) of the subject’s operating characteristic curve (ROC). The AUC with superior discriminative ability is 1.0, and the AUC without discriminative ability is 0.5. To comprehensively consider the performance of machine learning classifiers, we also used the metrics Accuracy, Sensitivity, Specificity, Precision and F1-score for evaluation, defined as

5 $$\begin{aligned} Accuracy= & {} (TP+TN)/(TP+FP+FN+TN) \end{aligned}$$

6 $$\begin{aligned} Precision= & {} TP/(TP+FP) \end{aligned}$$

7 $$\begin{aligned} Sensitivity= & {} TP/(TP+FN) \end{aligned}$$

8 $$\begin{aligned} Specificity= & {} TN/(TN+FP) \end{aligned}$$

9 $$\begin{aligned} F1\_score= & {} 2*(Precision*Sensitivity)/(Precision+Sensitivity) \end{aligned}$$

where TP (true positive), TN (true negative), FP (false positive) and FN (false negative) are the values in the confusion matrix, and each final result was subjected to multiplicative cross-validation. Then we took their mean and standard deviation.

## Data Availability

The data that support the findings of this study are available from Nanfang Hospital, Southern Medical University but restrictions apply to the availability of these data, which were used under license for the current study, and so are not publicly available. Data are however available from the authors upon reasonable request and with permission of Nanfang Hospital, Southern Medical University.
